# The evidence base for professional and self-care prevention - caries, erosion and sensitivity

**DOI:** 10.1186/1472-6831-15-S1-S4

**Published:** 2015-09-15

**Authors:** Svante Twetman

**Affiliations:** 1 Department of Odontology, Faculty of Health and Medical Sciences, University of Copenhagen, Copenhagen, Denmark

**Keywords:** antibacterial agents, fissure sealant, fluoride, remineralizing agents, systematic reviews

## Abstract

**Background:**

The aim of this conference paper was to examine the evidence base for primary and secondary prevention of dental caries, erosions and dentin hypersensitivity through professional and self-care measures.

**Methods:**

A mapping of systematic reviews (SR) of literature was carried out in PubMed and the Cochrane library through April 2014 using established MeSH-terms and disease-related search words in various combinations. The search was restricted to SR's published in English or Scandinavian and all age groups were considered. The reference lists of the selected papers were hand-searched for additional review articles of potential interest. Meta-analyses, guidelines and treatment recommendations were considered only when SR's were lacking. In the event of updates or multiple systematic reviews covering the same topic, only the most recent article was included. No quality assessment of the systematic reviews was carried out. The quality of evidence was rated in four levels according to the GRADE scale.

**Results:**

In total, 39 SR were included. For primary caries prevention, the quality of evidence was high for the use of fluoride toothpaste (with and without triclosan) and moderate for fluoride varnish and fissure sealants. The quality of evidence for fluoride gel, fluoride mouth rinse, xylitol gums and silver diamine fluoride (SDF) was rated as low. For secondary caries prevention and caries arrest, only fluoride interventions and SDF proved consistent benefits, although the quality of evidence was low. Likewise, the GRADE score for preventing erosions located in the enamel with fluoride supplements was low. The quality of evidence for various professional and self-care methods to prevent and manage dentine hypersensitivity was very low.

**Conclusions:**

There are knowledge gaps in many domains of cariology and preventive dentistry that must be addressed and bridged through clinical research of good quality.

## Introduction

Dental caries, dental erosion and dentin hypersensitivity are prevalent oral conditions occurring at all ages. They are all multifactorial; caries is a biofilm-mediated disease resulting from a complex interaction between the commensal microbiota, host susceptibility and environmental factors such as diet [1], dental erosion is the non-reversible loss of enamel and dentin when exposed to non-bacterial extrinsic or intrinsic acids [2] and dentin hypersensitivity is a short sharp pain arising from exposed dentine in response to stimuli [3]. The three conditions also share the feature of being largely preventable and there are numerous primary research papers and narrative reviews available on the effectiveness of various methods to reduce the burden of disease. However, in the current era of evidence-based dentistry and care, systematic reviews and meta-analyses, based on randomised controlled trials, are considered top of the hierarchy concerning efficacy of preventive interventions and therapies. The aim of the present conference paper was therefore to examine and summarize the quality of evidence for primary and secondary prevention of dental caries, erosions and dentin hypersensitivity on the basis of current systematic reviews of literature. As the paper was focused on prevention in dental practice, community fluorides (water, milk, salt), public health programs or school-based interventions were not addressed.

## Methods

A broad search for systematic reviews of literature was carried out in PubMed (clinical queries) and in the Cochrane library through April 2014 using the main search words “caries”, “dental decay”, “early childhood caries”, “prevention”, “oral health promotion”, “antimicrobials”, “sugar”, “sugar substitutes”, “diet”, “fluoride”, “oral hygiene”, “dental erosion”, “dentin hypersensitivity” and “tooth sensitivity” in various combinations. Literature published in English, or in any of the Scandinavian languages, was considered and the reference lists of the selected reviews were hand-searched for additional review articles of potential interest. All age groups were of interest. If more than one systematic review on the same subject was identified, only the most recent date was included. However, no quality assessment of the systematic reviews was carried out. In the absence of systematic reviews, meta-analyses, guidelines and treatment recommendations were appraised, but only when based on a thorough and well-defined search strategy.

### 

#### Prevented fraction and quality of evidence

The caries prevented fraction (PF) was extracted from the systematic reviews when possible. PF, expressed as percent, was defined as the difference between caries increment in control group and the experimental group, divided by the increment in the control group. For dental erosions and sensitivity, no such clear-cut endpoint of efficacy could be found and a narrative synthesis was made. The quality of evidence was rated in four levels according to the GRADE scale [4]:

• High (⊕⊕⊕⊕). Based on high or moderate quality studies containing no factors that weaken the overall judgement. The true effect lies close to that of the estimate of the effect.

• Moderate (⊕⊕⊕O). Based on high or moderate quality studies containing isolated factors that weaken the overall judgement. The true effect is likely to be close to the estimate of the effect, but there is a possibility that it is substantially different.

• Low (⊕⊕OO). Based on high or moderate quality studies containing factors that weaken the overall judgement. The true effect may be substantially different from the estimate of the effect.

• Very low (⊕OOO). The evidence base is insufficient when scientific evidence is lacking, quality of available studies is poor or studies of similar quality are contradictory. Our confidence in the effect estimate is limited: The true effect is likely to be substantially different from the estimate of the effect.

In systematic reviews relying on other ratings or quality systems, the author's statements and conclusions were subjectively transformed to GRADE by the author of this paper: GRADE High=Strong; GRADE Moderate=medium, good; GRADE Low=limited; fair; GRADE Very Low=weak, insufficient, inconclusive; some evidence.

## Results

A total number of 39 systematic reviews were included to form the current evidence base; 24 were on primary caries prevention with various fluoride and non-fluoride agents, 4 dealt with secondary caries prevention, 3 compiled the evidence for prevention of erosions and 8 were about dentin hypersensitivity.

### Evidence base for caries prevention - general reflections

Dental caries is one of the most prevalent diseases on the globe. It is almost a paradox to find strong evidence for selected caries-preventive measures when a recent report has calculated that 2.5 billion people (35%) on the planet have untreated caries in their permanent dentition [5]. One obvious problem is that both the dental professionals and the dental diseases are unevenly distributed in communities, as is the ability and willingness to pay for care. The quality of evidence is generally stronger for primary prevention in the young permanent dentition than for secondary prevention, as elaborated below. This is a major concern in the era of non-invasive caries management. Likewise, the evidence for caries-preventive interventions in the primary dentition was generally weaker. Most clinical research has been conducted in children and adolescents, populations at risk or fragile elderly. This is another problem since caries is a life-long continuum and most cavities appear actually in adults today. Consequently, there are certain knowledge gaps for the efficacy of preventive technologies in non-compromised adults.

### Primary caries prevention

**Figure 1:**
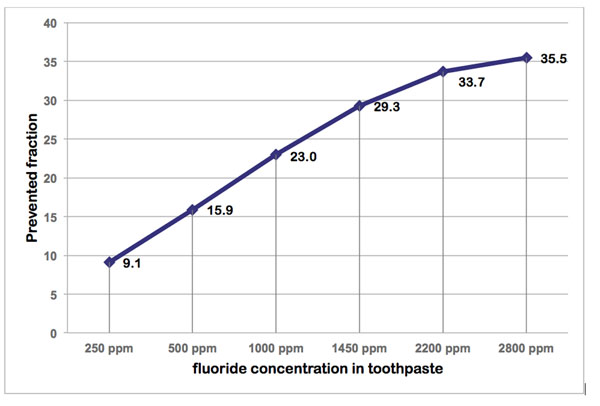
The caries-prevented fraction (PF) in relationship to fluoride concentration in toothpaste as compare with no fluoride. Data from Walsh et al. [8].

The prevented fraction and quality of evidence for the fluoride-based technologies are summarized in Table 1 [5-11]. Fluoride, in all forms, has an indisputable beneficial effect on caries at all ages [30]. The quality of evidence was rated high for daily use of fluoride toothpaste with a mean prevented fraction of 24% in permanent teeth in comparison with no fluoride. The PF was however clearly dose-dependent, ranging from 15.9% for toothpastes with 500ppm F to 35.5% in formulations with 2,800ppm F (Figure 1). For primary teeth, the quality of evidence was graded as low and notably, the caries-preventive effect from the low-fluoride formulas (<1000ppm) was statistically non-significant [31,32]. There was however high quality evidence showing that tooth brushing with fluoride toothpaste was more effective when carried out twice a day than irregular, and when supervised by a custodian during childhood [6,7]. No systematic review was identified concerning high-fluoride toothpastes (>2,800ppm F).

**Table 1:** Primary caries prevention: prevented fraction in permanent teeth versus placebo and quality of evidence for self-applied and professional fluorides

Method	Prevented fraction^a^	Quality of evidence^b^	Reference No.
**Self-care**			
Fluoride tooth paste	24%	⊕⊕⊕⊕	[6-8]
Fluoride mouth rinse	29%	⊕⊕OO	[9]
Fluoride tablets, drops, lozenges, gums	24%	⊕OOO^c^	[10]
			
**Professional care**			
Fluoride varnish	43%	⊕⊕⊕O	[11]
Fluoride gel	21%	⊕⊕OO	[6]

^a^Prevented fraction = difference in caries increment between a test and a control group, divided by the mean caries increment in the control group.

^b^according to the GRADE-scale:

• High (⊕⊕⊕⊕). Based on high or moderate quality studies containing no factors that weaken the overall judgement. The true effect lies close to that of the estimate of the effect

• Moderate (⊕⊕⊕O). Based on high or moderate quality studies containing isolated factors that weaken the overall judgement. The true effect is likely to be close to the estimate of the effect, but there is a possibility that it is substantially different

• Low (⊕⊕OO). Based on high or moderate quality studies containing factors that weaken the overall judgement. The true effect may be substantially different from the estimate of the effect

• Very low (⊕OOO). The evidence base is insufficient when scientific evidence is lacking, quality of available studies is poor or studies of similar quality are contradictory. Our confidence in the effect estimate is limited: The true effect is likely to be substantially different from the estimate of the effect

The runner-up was fluoride varnish with a moderate quality of evidence and a PF of 43% following 2-4 professional applications per year in permanent teeth compared with placebo/no treatment. The prevented fraction seemed to be higher in vulnerable populations compared to populations with low caries risk. Concerning primary teeth, the caries preventive effect of fluoride varnish was ambiguous. Most studies were in favor for professional applications in vulnerable preschool populations (PF18-24%), but displayed an unclear risk of bias [33,34]. The quality of evidence was also graded as low for fluoride mouth rinse (FMR) and professionally applied fluoride (APF) gels in in children and adolescents. Both methods may however be beneficial in high-risk populations with irregular use of fluoride toothpaste and in individual subjects with increased caries risk (e.g. fixed orthodontic appliances). The evidence base for fluoride supplements (tablets, lozenges, drops and chewing gums) was rated very low, mainly due to a lack of clinical trials with moderate or low risk of bias.

The quality of evidence concerning the non-fluoride methods for professional and self-care caries prevention is shown in Table 2 [12-29]. The best non-fluoride measure to prevent fissure caries in newly erupted permanent molars was without doubt fissure sealants with resin-based materials. The quality of evidence was rated as moderate for high-risk children but the information on the magnitude of benefit in low-caries populations was scarce and the relative effectiveness of different types of sealants has yet to be established. Furthermore, the method is costly and requires regular check-ups and repair in order to maintain a high prevented fraction. The evidence for use of xylitol in gums, tablets and candy was rated as low, with the daily amount needed, costs and compliance as obvious obstacles for its large scale use. The quality of evidence for the antibacterial technologies (chlorhexidine, ozone), interdental cleaning and oral health promotion were rated as very low, in most cases due inconsistent or conflicting findings in studies of equal quality, or lack of randomized controlled trials. Moreover, the role of dietary counselling in caries prevention proved unclear. One recent systematic review presented moderate quality of evidence that dietary sugar restriction was related to a reduction in caries [36] while another linked the frequency of sugar intake rather than the amount to caries frequency [37]. The findings in both reviews were however largely based on older observational studies and the evidence for one-to-one dietary interventions in the dental setting was rated as very low according to Harris and co-workers [26].

**Table 2:** Primary caries prevention: prevented fraction in permanent teeth and quality of evidence for non-fluoride measures

Method	Prevented fraction^a^	Quality of evidence^b^	Reference No.
**Self-care**			
Xylitol gums	59%	⊕⊕OO	[12,13]
Triclosan/copolymer toothpaste	5%	⊕⊕⊕⊕	14
Interdental cleaning (brushing and flossing)	24%	⊕OOO	[15,16]
			
**Professional care**			
Fissure sealants	84-65%c	⊕⊕⊕O	[17,18]
Chlorhexidine varnish	21%	⊕OOO	[19,20]
Silver diamine fluoride (38%)	70%	⊕⊕OO	[21,22]
Ozone	NDd	⊕OOO	[23]
Oral health counselling, motivational interviews	ND	⊕OOO	[24,25]
Dietary intervention	ND	⊕OOO	[26]
Early age preventive dental visits, Recall intervals	ND	⊕OOO	[27-29]

^a^ prevented fraction = difference in caries increment between a test and a control group, divided by the mean caries increment in the control group

^b^ according to the GRADE-scale, see Table 1.

^c^ 2-9 year follow-up

^d^ ND = not determined?

In recent years, the addition of antibacterial (triclosan) or pH-rising (arginine) supplements to fluoride toothpastes has been introduced. There was evidence of high quality that triclosan and copolymers slightly may increase the caries preventive effect on coronal caries (PF 5%) in comparison with standard fluoride toothpaste while the support to reduce root caries was weaker [14]. No systematic review could be identified concerning the novel pro-arginine-system.

### Secondary caries prevention

As stated above, there are few systematic reviews available on secondary caries prevention or caries control, which means re-mineralization or arrestment of existing early, non-cavitated lesions. The present quality of evidence is presented in Table 3 [38-41]. The unsolicited recent systematic review by Tellez and co-workers [38] included 29 primary studies on the topic but only 10 had moderate or low risk of bias. It was concluded, with a low quality of evidence, that fluoride interventions (toothpastes, varnishes, mouth rinses, gels) had the most consistent benefit in decreasing the progression of non-cavitated enamel lesions and, especially, in arresting root caries lesions. Likewise, studies on sealants suggested a potential benefit in the arrestment of pre-cavitated fissure caries. Studies on xylitol and chlorhexidine were limited in number and, in the majority of the cases, did not show a statistically significant effect on caries incidence. Several re-mineralizing agents have been introduced in recent years to supplement or enhance the effect of fluoride, of which the calcium phosphopetide-amorphous calcium phosphate (CPP-ACP) concept is most studied *in vivo*. Although findings suggested CPP-ACP to work better than placebo on early enamel lesions, the effect was not significantly different from that of fluoride. The quality of evidence, based on current clinical trials, was graded as very low [40].

**Table 3:** Quality of evidence for secondary crown and root caries prevention (remineralization of early lesions, caries control, caries arrest)

Technology	Quality of evidence^a^	Reference No.
Fluoride interventions^b^	⊕⊕OO	[38,39]
Xylitol (lozenges, chewing gums, tablets)	⊕OOO	[38]
Chlorhexidine varnish	⊕OOO	[20]
CPP-ACP (toothpaste, mouth rinse, tooth mousse, chewing gum)	⊕OOO	[40]
Silver diamine fluoride	⊕⊕OO	[21,22]
Ozone	⊕OOO	[41]
Resin sealants	⊕OOO	[38]

^a^ according to the GRADE-scale, see Table 1.

^b^ toothpaste, varnish, mouth rinse, gel

The use of 38% antibacterial silver diamine fluoride (SDF) is controversial in secondary caries prevention. Two available systematic reviews [21,22] were based on a small number of primary trials but report consistently a high prevented fraction (56-100%) for caries arrest, both in the primary and permanent dentition. However, there are methodological concerns as there is no validated way to measure caries arrest in an objective way. The quality of evidence was rated as low but promising, especially for very in young children unable to cooperate with conventional treatment or for patients with special need. No significant complications have been reported in association with SDF applications but a black staining of carious lesions always occurs and a mild gingival irritation may occur. Furthermore, SDF remains non-approved by dental organizations in many countries due to toxic and environmental concerns.

The relative knowledge gap for secondary prevention is a drawback because the early “whitish”, demineralized enamel with rough structure is often the first detectable clinical sign of caries activity, calling for individualized and targeted prevention. Thus, and needless to say, there is an urgent need of high-quality clinical research on caries lesion control to elucidate this topic.

## Evidence for prevention of erosion

Dental erosion is the progressive and irreversible loss of dental hard tissue caused by acids from non-bacterial compounds. Consequently, the primary prevention of this condition would simply be to avoid any contact with erosive drinks and natural acid or acid-flavored food items. When eating-disorders, vomiting or gastric reflux are parts of the risk complex, psychological counselling, lifestyle changes and medication are essential components for the management of the condition. Dentists are however more commonly facing already established dental erosions, or tooth wear, of various stages and being an irreversible condition, there is an indistinct zone between secondary prevention and therapy. There are several systematic reviews available on the prevalence and etiology of dental erosions but very few dealing with its prevention or management. Among the non-invasive preventive measures to arrest this chemical process, diet changes, water rinses, fluoride-containing products, calcium-additives, matrix metalloproteinases and laser therapy have been commonly suggested but hardly evaluated in a scientific way. A recent systematic review [42] has graded the quality of evidence for fluoride varnish and fluoride toothpaste as well as calcium added to fruit juices as very low, albeit fluoride-containing agents may be helpful in decreasing sensitivity [2]. A second review, based on three included *in situ* studies, displayed low quality evidence that fluoride may have a re-mineralizing role to play in reducing dental erosions, when located in the enamel [43]. No evidence relating the effectiveness of different strategies to apply dietary advice for the prevention of dental erosion was identified in a Cochrane review [44]. The use of low-abrasive toothpastes, re-mineralizing agents and bonding agents applied to exposed dentin are frequently advocated measures in the literature but not supported by extensive research. To establish a solid base of evidence, there is a need to perform randomized controlled trials on large and compatible study groups. In this context, a diagnostic device for monitoring longitudinal erosive changes would be helpful.

## Evidence base for prevention of dentin hypersensitivity

The primary preventive measures for dentin hypersensitivity are common-sense based and almost identical to those advocated for dental erosions; avoid erosive (acidic) drinks and dietary sources and execute gentle but efficient tooth brushing with low abrasive toothpaste, separated from the acid intake. There are however no systematic reviews covering such counselling. For dental professionals, obvious measures to prevent the risk of tooth sensitivity is to avoid multiple in-office bleaching sessions with prolonged applications of high-concentrated hydrogen peroxide and to make gap-free fillings. For the management of established dentin hypersensitivity, treatment is perhaps a more adequate term than prevention. There are two main approaches to treat hypersensitive teeth [45]; i) blocking of the exposed dentin tubules to prevent fluid movements, and, ii) inhibition of the neuronal transmission of the stimuli. The first mechanism is employed by the majority of desensitizing agents currently available on the market, including fluoride varnishes, oxalate gels, dentin bonding and laser therapy. The second mechanism is based on application of potassium nitrate as typical example. In addition, surgical root coverage may be an option for the treatment of cervical root exposure. The evidence base for various treatment methods is summarized in Table 4 [46-52]. The body of good and rigorously controlled studies to demonstrate clinical efficiency in hypersensitivity reduction was however not large; most primary studies employed small and diverse study groups and displayed a variety of designs and application modes. Pain was provoked by tactile, evaporative or thermal stimuli and commonly subjectively measured via verbal or numerical descriptors. Thus, the effect-size from one study could not be compared with another and not readily synthesized. The quality of evidence was rated as very low for all technologies which do not mean that all of them were ineffective. Multiple approaches to plug open tubules appear to alleviate pain but no universally accepted agent or treatment has been identified. Two systematic reviews, comparing laser therapy with desensitizing agents, suggested that lasers seemed to have a slight clinical and immediate advantage over topically applied medicaments [51,52]. However, as conflicting findings appear frequently following both self-applied and professional methods, research can currently not proclaim one technique to be superior to another in the management of dentin hypersensitivity.

**Table 4:** Evidence base for management of dentin hypersensitivity

Strategy	Example of agents	Quality of evidence^a^	Reference No.
**Self-care**			
Impede neural transmission	Potassium nitrate	⊕OOO	[46]
Blocking tubules	Oxalate, arginine	⊕OOO	[47,48]
Blocking tubules	Fluoride-, calcium-, strontium-salts, SnF	⊕OOO	[3]
**Professional care**			
Blocking tubules	Fluoride varnish, gel	⊕OOO	[46]
Dentin sealers	Resin, adhesives	⊕OOO	[49]
Lasers	GaLAS, Nd:YAG, ErYAG/Co2	⊕OOO	[50,51]
Surgical	Root coverage	⊕OOO	[52]

^a^according to the GRADE-scale, see Table1.

## Discussion

Mapping of systematic reviews offer is an established transparent way to describe research within a field and to identify gaps in the research base [53]. The process is however not without problems; for example, the lack of standardization concerning the quality assessment is a key issue. In this paper, criteria for quality that appeared in the various reviews were subjectively transformed by a single author to GRADE terms. Obviously, this was a certain shortcoming and may have introduced bias in the reporting of results.

At a first glance, it may be disappointing to find that so much of the preventive care relies on low or very low quality of evidence. However, this is not the same as saying “do not do” because evidence-based dentistry is not a cook-book approach to clinical dentistry. A certain intervention may be useful even though the evidence is weak or even lacking. The quality grade “very low” is in fact in most cases a knowledge gap due to a shortage of good primary studies or, non-existent studies. The quality of evidence should exclusively be based on relevant, scientifically sound studies with low or moderate risk of bias. Treatment recommendations and guidelines on the other hand, are a result of merging the evidence with clinical experience, expert opinions and good clinical practice. This means that a technology rated as “very low” might work well in a skilled hand and under certain conditions. At contrary, methods with high quality of evidence might be of no or very small clinical importance [14]. Furthermore, while evidence is global, guidelines and recommendations must be locally adopted to national or even regional conditions and circumstances. And, at the end of the day, a method is never better than its compliance and/or the patients’ willingness to pay.

A shortcoming with the present paper was the language restriction and that only one major database was searched and therefore, relevant systematic reviews may have been missed. Another limitation was that no structured quality assessment of the systematic reviews was conducted, for example with the AMSTAR-tool [54]. Factors that can weaken the quality of evidence are study limitations, indirectness, inconsistency, imprecision and publication bias. Obviously, the methodological quality of the included systematic reviews varied and a common drawback was that each primary study was not given an overall assessment of its risk of bias. Another frequent issue was that results from studies with high risk of bias were pooled and synthesized with the better ones, which, in turn, significantly can influence the effect size. There is therefore a risk that the calculated prevented fraction for the various technologies may be either over- or underestimated, especially in cases when few primary studies were accepted. In any case, the need for better evidence remains.

A striking fact was that health-economic evaluations and ethical considerations in connection with primary and secondary prevention were almost non-existent in the systematic reviews [55] and patient-oriented aspects, such as acceptability of an intervention, were only occasionally addressed. This should however not be used as an excuse not to select one technology before another. For example, methods that may expose patients to a risk should be avoided as well as methods involving particularly high costs until they have been proven superior or at least “as good as” in clinical trials. In other words, in the absence of evidence for novel or alternative technologies, it is safer to adhere to establish methods. In this context, a pertinent question is if the dental professionals really utilize what is already known? As shown above, there is strong scientific evidence for fluoride toothpaste as the best way of preventing and controlling caries at all ages. Toothpastes are self-applied, affordable, paid by the patients with clear guidelines on its use. Yet, epidemiological surveys indicate that approximately 30% of all patients brush their teeth on an irregular non-daily basis. Even worse, only 10% report that they use fluoride toothpaste in an “optimal” way according to criteria established within the research team: a) two times a day, b) 2 minutes, c) apply a full brush, and d) do not rinse afterwards [56]. A recent questionnaire among patients of various age groups indicated that patients actually weren't told how to use fluoride and a qualitative study unveiled that dental professionals “took for granted that patients knew” [56,57]. Consequently, an important step in preventive practice is not only to focus on what to say, based on best available evidence, but also improve the skills on how to say it and make sure that the message is understood by the patients.

In conclusion, there was high and moderate quality of evidence for the use of fluoride technologies and fissure sealants for primary prevention of dental caries in the young permanent dentition. For secondary caries prevention, the quality of evidence was rated as low or very low. In elderly, systematic reviews of literature were unable to establish definite conclusions regarding the effectiveness of adjunct methods for caries prevention. The quality of evidence for prevention of dental erosions and dentin hypersensitivity was very low. Thus, there is an urgent need of clinical research of good quality in many domains of cariology and preventive dentistry.

## Declaration of interests

The author received funding from Colgate Palmolive to attend and present at the “Prevention in Practice” conference. This was provided in the form of flights, hotel accommodation and subsistence. No one from the Colgate Palmolive Company was involved in the production, assessment or peer review of the manuscript nor was it submitted to them for approval prior to publication.

## Declarations

This article has been published as part of *BMC Health Services Research* Volume 15 Supplement 1, 2015: Proceedings of Prevention in practice - making it happen. The full contents of the supplement are available online at http://www.biomedcentral.com/bmchealthservres/supplements/15/S1. Publication charges for this supplement were funded by Colgate Palmolive.
